# P-1779. Effect of a Transitions of Care Pharmacist Intervention on Discharge Oral Antibiotic Prescribing

**DOI:** 10.1093/ofid/ofae631.1942

**Published:** 2025-01-29

**Authors:** Stephen Argiro, Jason Hedvat, Michaela L Myerson

**Affiliations:** NYU Langone Health, New York; Mount Sinai Beth Israel, New York, New York; Mount Sinai Beth Israel, New York, New York

## Abstract

**Background:**

An emerging area of focus in antibiotic stewardship programs (ASP) is appropriate antibiotic selection at hospital discharge. The purpose of this study was to assess the effect of a transitions of care (ToC) pharmacist-led review of discharge oral antibiotic therapy on antibiotic appropriateness.

**Methods:**

This was a prospective single center case-control study at Mount Sinai Beth Israel that included adult patients (≥18 years old) discharged on oral antibiotics for the treatment of common infectious diseases syndromes between February 13, 2023 and May 13, 2023. Appropriateness of antibiotic regimen was based on standardized guidance developed by the ASP team. The primary outcome was the difference in percentage of inappropriate antibiotic prescriptions between the provider-only and ToC pharmacist-reviewed groups. Secondary outcomes included mean duration of therapy between the two groups, percentage of pharmacist interventions accepted, and number of fluoroquinolone prescriptions avoided. Comparisons were performed using Chi-squared or Fischer’s exact test for categorical variables and Student’s t test or the Mann-Whitney U test for continuous variables, as appropriate. P < .05 was considered statistically significant.

**Results:**

A total of 182 patients were included in the analysis, 81 patients in the provider-only group and 101 patients in the ToC pharmacist-reviewed group. The most common diagnoses were intra-abdominal infection (37.9%), urinary tract infection (23.1%), skin and soft tissue infection (21.4%), and community acquired pneumonia (17.6%). Patients in the ToC pharmacist-reviewed group were significantly less likely to have an inappropriate antibiotic prescription at discharge when compared to the provider-only group (25.7% vs. 87.7%; p < .001). The mean duration of therapy was significantly lower in the ToC pharmacist-reviewed group when compared to the provider-only group (7.7 vs. 9.1 days; p =.007). In the ToC pharmacist-reviewed group, 56.9% (33/58) of pharmacist interventions were accepted and 19.3% (11/57) of fluoroquinolone prescriptions were avoided.

**Conclusion:**

A ToC pharmacist’s review of oral antibiotics at discharge significantly reduced inappropriate antibiotic selection and duration of therapy.

**Disclosures:**

**All Authors**: No reported disclosures

